# 

**Published:** 1972-04

**Authors:** 


					,
BRISTOI
MEDICO
CHIRURGICAL
JOURNAL
:m|IONAL LIBRARY OF MEDICINE
INDEX COPY
OQJ 25 1972
.IMP^ NUV i ' 1972
?i j|
?? &?' ' IH'IIIH m  ?*"*
a^ril
1972
VOL. 87 (ii) No. 322

				

